# Effects of the common 34C>T variant of the AMPD1 gene on immune function, multiorgan dysfunction and mortality in patients with sepsis

**DOI:** 10.1186/cc11955

**Published:** 2013-03-19

**Authors:** B Ramakers, E Giamarellos-Bourboulis, M Coenen, M Kox, J Van der Hoeven, C Routsi, A Savva, I Perdios, F Diamantea, D Sinapidis, P Smits, N Riksen, P Pickkers

**Affiliations:** 1 Radboud University Nijmegen Medical Centre, Nijmegen, the Netherlands; 2 University of Athens, Medical School, Athens, Greece; 3 Nafplion General Hospital, Nafplio, Greece; 4 'G.Gennimatas' General Hospital, Athens, Greece; 5 Alexandra General Hospital, Athens, Greece

## Introduction

Adenosine exerts anti-inflammatory and tissue protective effects during systemic inflammation. While the anti-inflammatory properties may induce immunoparalysis and impede bacterial clearance, the tissue protective effects might limit organ damage. The effects of a common loss-of-function variant of the adenosine monophosphate deaminase 1 gene (AMPD1), which is associated with increased adenosine formation, in patients with sepsis are unknown.

## Methods

In a prospective cohort, genetic-association study, the effects of the presence of the AMPD1 gene on immune function, multiorgan dysfunction and mortality in septic patients was studied. Pneumosepsis patients (*n *= 402) and controls without infection (*n *= 101) were enrolled.

## Results

**Figure 1:**
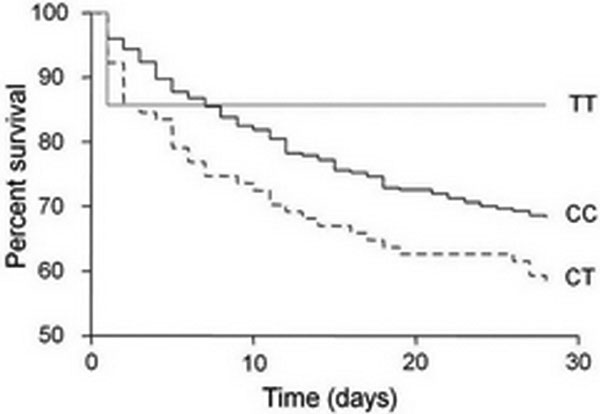
**Kaplan-Meier curve for the 402 sepsis patients**.

In pneumosepsis patients and controls, a similar prevalence of the 34C>T (rs17602729) mutation in the AMPD1 gene was found. Univariate logistic regression analysis showed a tendency of increased mortality in patients with the CT genotype, compared with patients with the CC genotype (OR 1.53; 95% CI 0.95 to 2.5). Moreover, carriers of the CT genotype tended to suffer more from multiorgan dysfunction, OR 1.4 (0.84 to 2.3) and 3.0 (0.66 to 13.8), for CT and TT, respectively (*P *= 0.07). In septic carriers of the CT genotype, the *ex vivo *production of TNFα by LPS-stimulated monocytes was attenuated (*P *= 0.005), indicative for more pronounced immunoparalysis in these patients. See Figure 1.

## Conclusion

The presence the 34C>T variant of the AMPD1gene is not related to infection susceptibility; however, it is associated with more pronounced immunoparalysis in patients with sepsis, and shows a tendency towards increased mortality. Mechanistically, the anti-inflammatory effects of adenosine may account for this and apparently overrule its tissue protective effects.

